# *De Novo* Assembly and Annotation of the Complete Genome Sequence of Myxococcus xanthus DZ2

**DOI:** 10.1128/mra.01074-21

**Published:** 2022-04-06

**Authors:** Rodolfo Aramayo, Beiyan Nan

**Affiliations:** a Department of Biology, Texas A&M University, College Station, Texas, USA; Montana State University

## Abstract

We report the assembly and annotation of a high-quality genome sequence for Myxococcus xanthus strain DZ2 (GenBank accession number CP080538), created using a combination of short reads generated using DNBSEQ technology (BGI Genomics) and long high-fidelity (HiFi) reads generated using Pacific Biosciences (PacBio) technology.

## ANNOUNCEMENT

The Myxococcus xanthus isolate (GenBank accession number CP080538) reported here was originally acquired by David Zusman from the Roger Stanier collection at UC Berkeley and named DZ2 (1). While this work was in progress, Jain et al. (2) reported the assembly of a Myxococcus xanthus DZ2 isolate of similar origin (CP070500). While the assembly submitted under GenBank accession number CP070500 represents a big improvement over the previously reported draft DZ2 assembly (3), the assembly reported here (CP080538) is both larger in size and different in gene content (Table 1).

**TABLE 1:** Comparative analysis of the highly related Myxococcus xanthus strain DZ2 assemblies

Characteristic^*a*^	Data for assembly (GenBank accession no.):	
	CP080538	CP070500
Genome size (bp)	9,365,783	9,359,382
Total no. of genes	7,581	7,576
Total no. of CDSs	7,499	7,494
No. of genes (coding)	7,402	7,408
No. of CDSs (with protein)	7,402	7,408
No. of RNA genes	82	82
No. of complete rRNAs (5S, 16S, 23S)	4, 4, 4	4, 4, 4
No. of tRNAs	66	66
No. of ncRNAs	4	4
No. of CDSs (without protein)	97	86
No. of pseudogenes:		
Total	97	86
With ambiguous residues	0	0
Frameshifted	49	37
Incomplete	52	56
With an internal stop	14	12
With multiple problems	15	16
No. of CRISPR arrays	4	4

a CDS, coding DNA sequences; ncRNAs, noncoding RNAs; rRNAs, ribosomal RNAs; tRNAs, transfer RNAs.

Cells of Myxococcus xanthus strain DZ2, derived from frozen stock from the Roger Stanier collection at UC Berkeley, were grown in liquid CYE medium (10 g/L Casitone and 5 g/L yeast extract), 8 mM MgSO_4_, and 10 mM 3-(*N*-morpholino)propanesulfonic acid (MOPS), pH 7.6, at 32°C, harvested by centrifugation, and frozen in liquid nitrogen. The pellet was ground into a fine powder, and DNA was extracted by lysing the cells with cetyltrimethylammonium bromide (CTAB) at 65°C. The DNA was purified using phenol/chloroform/isoamyl alcohol, followed by ethanol precipitation. DNA sequencing was performed by BGI Genomics using two different technologies: DNBSEQ and high-fidelity (HiFi) PacBio sequencing. Construction of the DNA libraries, DNA sequencing, and quality control (QC) of the long HiFi reads and the short reads derived from PCR-free rolling circle replication of the DNA nanoballs were all performed by BGI Genomics using their standard operating procedures (SOP) (4).

Sequencing generated a total of 436,127 HiFi PacBio subreads with an average length of 9,018 bp, totaling 3,933.4 Mbp (representing 420-fold genome coverage), and ∼10.2 million 100-bp paired-end DNBSEQ reads, totaling 2,042.54 Mbp (representing 218-fold coverage). Small reads that passed our standard quality control (QC) protocol (5) and large reads with a quality value (QV) of >20 or >99% accuracy were used for assembly. Genome assembly was performed using HiCanu version 2.1.1-Java-1.8 (6), Unicycler version 0.4.8 (7), and Velvet version 1.2.10 (8, 9). We performed a total of 48 assemblies (36 HiCanu, 11 Unicycler, and 1 Velvet). The parameters used and the associated resulting data are described in a supplemental Zenodo repository (10). The largest circular contig of the HiCanu assemblies (contig tig00000005 of Assembly 33), was ∼23.8 kbp larger than the largest Unicycler assembly and was thus selected for analysis. We then used PSI-CD-HIT version 4.8.1/blastn version 2.12.0+ (11–14) to verify that the smaller homologous contigs generated by the other assemblies were contained within this largest HiCanu assembly (10). Given that all the smaller homologous contigs were indeed contained within the largest HiCanu contig (tig00000005), this contig was then declared as our genome assembly and submitted to the NCBI databases (10). Genome annotation was performed using the NCBI Prokaryotic Genome Annotation Pipeline (PGAP) version 5.2 (15). The CP080538 assembly presented here differs from the CP070500 assembly in many ways. The CP080538 assembly is 6.4 kbp larger than the CP070500 assembly, and while it has five more genes and 11 more pseudogenes, it also has six fewer coding sequences (CDSs). Both assemblies have the same number of rRNAs, tRNAs, ncRNAs, and CRISPR arrays (Table 1).

### Data availability.

The raw data associated with this publication have been deposited at NCBI under BioProject accession number PRJNA748417 and SRA accession numbers SRX11508707 (PacBio reads) and SRX11508706 (Illumina reads). The complete genome sequence has been deposited in GenBank under accession number CP080538.1. The different genome assembly commands and resulting assembly files generated throughout this work have all been deposited at Zenodo (10).
